# Influence of Analog and Digital Crease Lines on Mechanical Parameters of Corrugated Board and Packaging

**DOI:** 10.3390/s22134800

**Published:** 2022-06-25

**Authors:** Tomasz Garbowski, Tomasz Gajewski, Anna Knitter-Piątkowska

**Affiliations:** 1 Department of Biosystems Engineering, Poznan University of Life Sciences, Wojska Polskiego 50, 60-627 Poznan, Poland; 2 Institute of Structural Analysis, Poznan University of Technology, Piotrowo 5, 60-965 Poznan, Poland; tomasz.gajewski@put.poznan.pl (T.G.); anna.knitter-piatkowska@put.poznan.pl (A.K.-P.)

**Keywords:** corrugated board, digital crease, analog crease, edge crush test, shear stiffness test, transverse shear test, bending stiffness, box compression test, finite element model

## Abstract

When producing packaging from corrugated board, material weakening often occurs both during the die-cutting process and during printing. While the analog lamination and/or printing processes that degrade material can be easily replaced with a digital approach, the die-cutting process remains overwhelmingly analog. Recently, new innovative technologies have emerged that have begun to replace or at least supplement old techniques. This paper presents the results of laboratory tests on corrugated board and packaging made using both analog and digital technologies. Cardboard samples with digital and analog creases are subject to various mechanical tests, which allows for an assessment of the impact of creases on the mechanical properties of the cardboard itself, as well as on the behavior of the packaging. It is proven that digital technology is not only more repeatable, but also weakens the structure of corrugated board to a much lesser extent than analog. An updated numerical model of boxes in compression tests is also discussed. The effect of the crushing of the material in the vicinity of the crease lines in the packaging arising during the analog and digital finishing processes is taken into account. The obtained enhanced computer simulation results closely reflect the experimental observations, which prove that the correct numerical analysis of corrugated cardboard packaging should be performed with the model taking into account the crushing.

## 1. Introduction

The environmental challenges faced by the contemporary world, such as an ecologically healthy lifestyle, thoughtful consumption of goods, and waste disposal in order to cause less damage to the environment, can be included in the three Rs, namely reduce, reuse, and recycle [1]. A global increase in the purchase of various products has been observed, however, great importance is attached to eco-friendly packaging. This requirement is also perfectly met by paperboard or corrugated board containers from the point of view of the three Rs principle mentioned above. Packages of this type are space saving after manufacturing, are easy to recycle, and are biodegradable. Furthermore, corrugated board is easy to print on (e.g., the brand or product name) and to shape by suitable creases. The execution of specially designed openings, ventilation holes, or perforations does not pose many problems. These features are of key importance with regard to shelf-ready packaging or retail-ready packaging in which the products are placed straight on the store shelves, ready to purchase. A solution such as this takes a significant amount of time and is a money saver for large sales companies. In the case of e-commerce, which at the present time, forms a substantial section of the sales market, individual customers may favor companies that deliver purchased goods with reusable packaging. In such cases, corrugated board is also an ideal choice.

However, it should not be forgotten that in light of all these features and requirements for cardboard packaging, it must first meet a certain load-bearing capacity, which is strictly related to the mechanical properties of paperboard/corrugated board. The producers of cardboard packaging constantly maintain a deep interest in effective, economical, and straightforward solutions, which, in turn, leads to the intensive development of scientific research closely related to this branch of industry.

Physical examinations are fundamental in assessing the strength of corrugated board boxes; therefore, a number of typical tests have been developed to standardize the process of characterizing the mechanical properties of corrugated board. The box compression test (BCT) [2,3,4,5,6] and edge crush test (ECT) [7,8,9,10,11] are the most prevalent. The behavior of the cardboard is inextricably linked to its dimensions: a high edge crush strength is crucial in low and stocky boxes, whereas in higher packaging the buckling strength, it is decisive [12,13,14]. The optimal selection of the constituents of corrugated board layers is of major importance for the load-bearing capacity of packages [15,16]. An option is the utilization of lighter boards as well as kraft or recycled papers, which have reduced mechanical parameters, saving material and thus diminishing cost. However, the condition of the guaranteed load capacity must be met.

To evaluate the strength of a corrugated board box, analytical formulae developed through the years and found in the literature can be utilized, starting from the simplest [17,18,19], used for typical box design, to the more complex approaches [2,3,4,20,21,22,23,24]. Renowned and widespread methods are also hybrid [2,3,4,25,26,27] or purely numerical approaches [28,29,30] using the finite element method [27,28,29,31,32,33,34]. Corrugated board is a layered structure with constituent layers made of paper, which is an anisotropic material, and thus performing numerical analysis is a demanding task because the material properties of each layer are required. The method of reducing a multilayer structure to a single layer is called homogenization. This process can be undertaken using analytical [35,36] or numerical approaches [15,30,37,38,39,40,41].

The basic raw materials used in the production of paper [42] are cellulose fibers, annual plants, and recycled materials, e.g., waste paper. Cardboard is made by alternately connecting layers of corrugated and flat paper [43]. The most commonly used connections range from two to seven layers. Fluting, i.e., paper for a corrugated layer, is formed by means of corrugating rollers, which give the desired shape and type of wave within strictly defined production parameters. The next step is to apply a binder, in this case starch glue, and a flat layer of paper, i.e., a liner. Joining subsequent layers is a duplication of the process. The profile of the corrugated web has high impact on the cardboard strength and is labeled by the letters A, B, C, E, and F. The difference between them is in flute height (A type is the tallest, F is the lowest), wavelength, and take-up factor, which is a quantification of the fluting length per unit length of the board [44]. For the most common packaging, cardboard with B and C flutes is applied; for big boxes, A fluting is used, and for the smallest, e.g., cosmetic packaging, E and F fluting is in use. In the case of double-wall corrugated board, different combinations of fluting are practiced, e.g., BC, BE, AE, FE, or EB, and vice versa. Due to the layered structure of the corrugated board, two characteristic in-plane directions of orthotropy associated with the mechanical strength of the paperboard can be distinguished: the machine direction (MD) perpendicular to the main axis of the fluting and parallel to the paperboard fiber alignment and cross direction (CD), which is parallel to the fluting.

The last stage of cardboard production is obtaining the appropriate sheet format. First, a cut along the sheet is performed and then lengthwise in a lateral direction. During this stage, it is also possible to crease (crush cardboard for bending). There are two possible variants of performing creases or die-cutting in the cardboard, namely analog [45,46] and digital [47,48]. Analog die-cutting is a technique suitable for large batches of boxes that consists of punching selected external shapes in paper and creating various intended holes. Punching can be combined with other techniques, e.g., concave/convex embossing, creasing, or perforation. The die has two kinds of knives (cutting and creasing knives) with specially profiled blades, which are driven into the cardboard with great force that causes damage to the structure. The damage to the corrugated board is usually around the creasing knives, because on both sides of the knives, rubbers are placed to prevent tearing of the liners while crushing the corrugated layers of a board.

Currently, the packaging design process does not take directly into account the effects of crushing the material; the most common concerns are with global correction (safety) factors. If, however, the decrease in the load capacity of the packaging due to the crushing of the creasing lines was carefully measured, the effect could be taken into account much more accurately in calculations. This would make the calculations dependent, for example, on the type of machine or type of finishing process. It is also possible to test some particularly important productions or series on weaker (and therefore cheaper) materials on digital devices, which crush the cardboard only to a small extent or not at all.

In the case of digital processes, die-cutting is performed using a laser while creasing is achieved either by means of specific laser cuts or a 3D-printed polymer on a replaceable carrier wound on a drum inside the finishing device. The former crease type enables control of the laser penetration into the corrugated board. However, due to material weakening (cutting the liners), it cannot always be applied to automatically folding boxes because the folding and gluing machines require a bending force within a certain predetermined range. The use of the latter type of creasing does not weaken the creasing lines but requires a preparation phase (printing the creasing rules), which, however, is still much faster and cheaper than preparing dies for conventional cutting tables. Both techniques are optimal for small and medium batches of boxes. The printing or coating of the paper has no influence on the digital process, although it is appropriate for cardboard up to the maximum B or C flute depending on the technique used.

This paper presents the results of laboratory tests on corrugated board and packaging produced using both analog and digital technologies. Cardboard specimens with digital and analog creases are subject to various mechanical tests [49], which allow for an assessment of the impact of creases on the mechanical properties of the cardboard itself, as well as of the behavior of the packaging. The ECT is employed to assess the compressive strength of corrugated board, and the load during the test is applied perpendicular to the axis of the flutes. In the bending stiffness test (BNT), four-point bending is performed; two supports are at the bottom of the cardboard while two equal forces operate on the specimen from the opposite side. The shear stiffness test (SST) involves twisting the cardboard cross-section by implementing a pair of forces at opposite corners while the other two remain supported. During the torsional stiffness test (TST), the cardboard sample is twisted in both directions. The ECT is standardized, and four different methods may be used, namely the edge clamping method [50], neck-down method [51], rectangular test specimen method [51,52,53], and the edge-reinforced method [54,55]. The differences in individual tests are due to the shape of the samples tested. All measuring techniques require that proper measurement methods and sensors be used to gather data. In the case of simultaneous force and displacement measurements, absolute encoders are often used, especially when the distance measurements must be highly accurate. In the tests conducted during the present research, an encoder with an accuracy of up to 5 μm was used.

For obtaining measurement data from the outer surfaces of the sample during testing, video extensometry can be applied. This procedure relies on measuring the relative distances between pairs of points tracked across images acquired at specific load values [8,56]. It is a similar but simpler method in comparison to digital image correlation (DIC), which, as a full-field noncontact optical measurement method, ensures high-level accuracy in data acquisition and is a huge advantage in the field of experimental mechanics. An application of DIC during the ECTs of damaged and undamaged panels made of corrugated paperboard was discussed in [57] and, for the BCT, in [14,58,59]. The results of combined compression and bending tests of paperboards and laminates for liquid containers while employing DIC are presented in [60,61].

In this paper, we compare the results of laboratory tests such as the ECT, SST, TST, and BNT to examine the impact of creases on the mechanical properties of cardboard and corrugated board boxes. The results allow for the preparation of an improved numerical model of the packaging in which all the observed and measured creasing effects in the area of the creasing line are taken into account, including those effects in the numerical analysis that allow us to obtain promising results.

## 2. Materials and Methods

### 2.1. Corrugated Board Testing

In this study, an innovative device for corrugated board testing was used, namely the BSE System [49]. The system was originally used for numerical estimations of box strength in which, based on various mechanical tests on corrugated board, finite element method computations were performed for particular box designs. In the present research, a BSE System testing device was used to characterize the mechanical properties of corrugated cardboards. In one Femat desktop device, several testing procedures were performed at the same time. The device was equipped with multiple sensors enclosed in one machine, which tracked the mechanical response of the cardboard samples. All data from the sensors were simultaneously sent to the main unit, which computed the representative mechanical parameters of the board tested using advanced inverse procedures. All force measurements are performed in the machine with strain gauges with an accuracy of up to 0.1 N, while the displacement measurement is performed with an absolute encoder with a resolution of 1 micrometer. In the following sections, we describe the testing protocols used.

#### 2.1.1. Edge Crush Test

The ECT is one of the most widely known mechanical testing methods used on corrugated board samples. It reflects the compressive behavior of the corrugated board in the cross-machine direction. There are multiple international standards for this testing method, although the general principle is similar, i.e., the sample of corrugated board of a particular size is placed along its cross-machine direction between rigid steel plates. An additional two steel blocks support the sample to hold it vertically, and the sample is statically compressed in a mechanical press.

**Figure 1:**
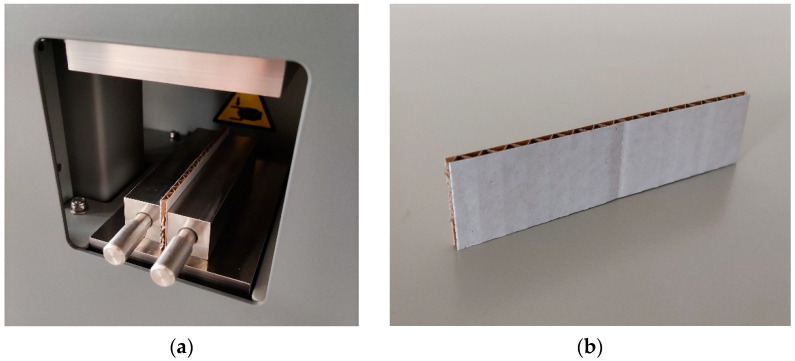
Edge crush test: (**a**) the testing socket and (**b**) the corrugated board sample.

The particular testing setup used for the ECT depends on the part of the world where the test is conducted. For instance, one may use ISO 3037, ISO 13821, FEFCO 8, or TAPPI T839. In this paper, the ISO 3037 standard was applied for ECTs using the BSE System from Femat [49]; see Figure 1a. The dimensions of the samples examined in this study were 25 × 100 mm; see Figure 1b.

#### 2.1.2. Shear Stiffness Test

The SST is an innovative mechanical testing method applied to corrugated board samples. It reflects the static transversal shear stiffness of the corrugated board tested and is able to capture the crushing of the board. In the second, updated generation of this testing method, the sample is square. The specimen is supported on two opposite corners and force is applied vertically at the other opposite corners.

**Figure 2:**
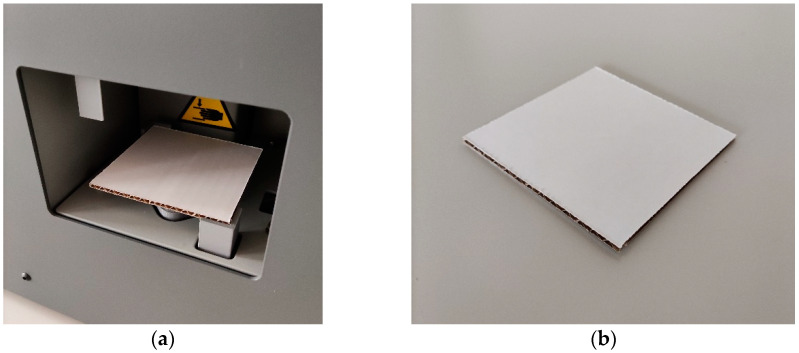
Shear stiffness test: (**a**) the testing socket and (**b**) the corrugated board sample.

There are no international standards, which regulate the testing setup for the SST. In this paper, the tests were performed by employing the BSE System from Femat [49]; see Figure 2a. The dimensions of the samples used here were 80 × 80 mm; see Figure 2b.

#### 2.1.3. Torsion Stiffness Test

The TST is an innovative mechanical testing method applied to corrugated board samples and reflects torsional stiffness. During the test, the longitudinal sample is clamped at the bottom and top and twisted statically by rotating the upper clamp around the direction of the sample. The test is performed along two directions of the cardboard, i.e., two samples must be cut from one material, in machine and cross-machine directions.

**Figure 3:**
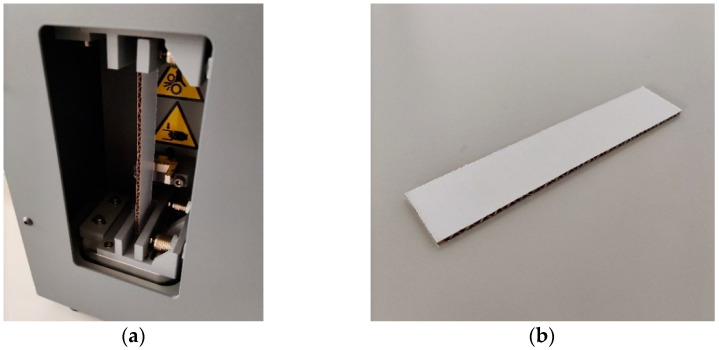
Torsion stiffness test: (**a**) the testing socket and (**b**) the corrugated board sample.

There are no international standards regulating the setup for the TST. In this paper, the tests were performed using the BSE System from Femat [49]; see Figure 3a. The dimensions of the samples used here were 25 × 150 mm; see Figure 3b.

#### 2.1.4. Bending Stiffness Test

The BNT is one of the most widely known mechanical testing methods used on corrugated board samples and reflects bending behavior. There are multiple international standards for this testing method. In this paper, four-point bending is considered, although the general principle in all examination standards is similar, i.e., the sample of corrugated board of a particular size is placed longitudinally with two perpendicular rigid supports. During the test, two upper perpendicular edges of the traverse beam statically press the sample and cause four-point bending. The test is performed along two directions of the cardboard, i.e., two samples must be cut from one material, in machine and cross-machine directions.

**Figure 4:**
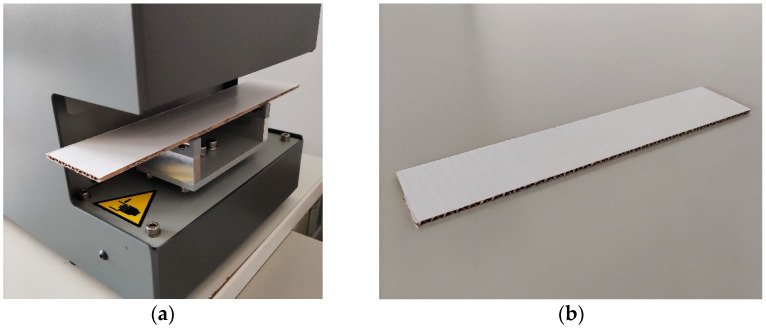
Bending stiffness test: (**a**) the testing socket and (**b**) the corrugated board sample.

The particular testing setup used for the BNT depends on the part of the world where the test is conducted. For instance, one may use ISO 5628 or TAPPI/ANSI T 836. In this paper, the ISO 5628 standard was used for four-point bending with the BSE System from Femat [49]; see Figure 4a. The dimensions of the samples used here were 50 × 250 mm; see Figure 4b.

### 2.2. Compression Test of Corrugated Board Packaging

The BCT is the most widely known mechanical testing method and reflects the compressive strength of a box. There are multiple international standards for this testing method, although the general principle is similar, i.e., the box sample is placed between two rigid steel plates and compressed by pressing the upper plate on the box.

**Figure 5:**
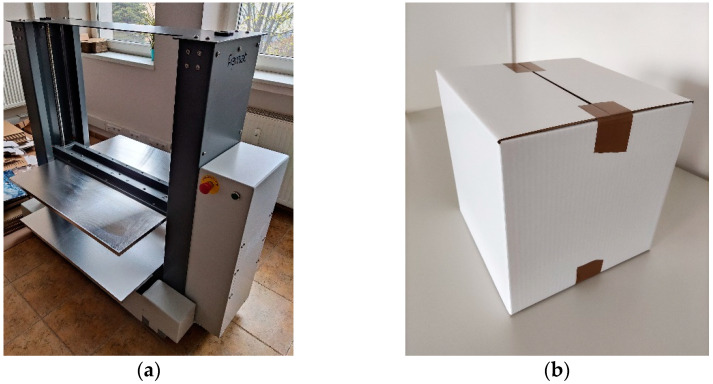
Box compression test: (**a**) the testing machine and (**b**) the box sample.

The international standards one may use for the BCT setup include ISO 12048, ASTM D642, or ASTM D4169-16. In this paper, the ISO 12,048 standard was adopted for the BCT by employing a mechanical press from Femat [49]; see Figure 5a. After production, the box samples were folded manually and taped to prevent the flaps opening during testing; see Figure 5b.

### 2.3. Analog and Digital Box Finishing Techniques

In this work, we used samples provided by a packaging manufacturer equipped with both packaging finishing technologies, namely analog and digital machines. The first is a traditional method based on flatbed die-cutting of packages and boxes. The technique involves cutting the boxes with a die to reproduce the designed shape of the packaging. The die has a knife with appropriately profiled blade shapes (different for cutting and creasing) and pressed with great force perpendicularly into the substrate (straight through). Lines of specific shapes can also be grooved, i.e., pressed into the surface. The die allows the cut packages to assume the desired external shapes, as well as to make holes and perforations. Rubber is often used around creasing knives to minimize the possibility of tearing the material. Unfortunately, the rubbers press on the material and can thus crush the cross-section of the corrugated board.

The newest methods of punching and creasing come down to a process based entirely on CO_2_ lasers [48] or hybrid solutions [47]. In digital finishing based on lasers, a crease is created as a result of specific laser cuts, which is used to facilitate the folding of the packaging along the cut lines (laser creasing). By definition, when cutting part of the way through the board, it necessarily affects its mechanical properties. Hybrid finishing is based on the patented Highcon digital adhesive rule technology. This method is based on 3D printing of creasing rules on a carrier that is mounted on a drum inside the finishing system and makes creases on the corrugated board immediately before cutting. Cutting, as in the previous method, is performed with a laser.

Samples prepared on analog and hybrid machines were made of the same materials. Therefore, it was possible to make an objective comparison of the two technologies and their influence not only on the material parameters of the corrugated board itself, but also on the strength of the packaging cut from those materials.

### 2.4. Corrugated Board and Box Samples

In the study, three single-walled corrugated boards of B flute were considered, henceforth labeled B360, B370, and B380.

The first corrugated board, B360, consists of the following papers:

● testliner 130 g/$m^{2}$;
● fluting 100 g/$m^{2}$;
● testliner 130 g/$m^{2}$.

The second corrugated board, B370, consists of the following papers:

● kraftliner brown 135 g/$m^{2}$;
● fluting 100 g/$m^{2}$;
● kraftliner brown 135 g/$m^{2}$.

The third corrugated board, B380, consists of the following papers:

● kraftliner white coated 145 g/$m^{2}$;
● fluting 100 g/$m^{2}$;
● duplex white 135 g/$m^{2}.$

Whether the delivered samples were made of the same material was first checked, and the actual grammage and thickness of all samples were measured (data are summarized in Table 1). It was concluded that in the case of the B360 and B370 models, the same materials were used, while in the case of the B380 model, the grammage of samples cut with the digital method was about 30 g lower than the grammage of samples cut using the analog method (despite the fact that the manufacturer’s designation indicated the same product). Most probably, for the production of both sets of samples, slightly different component papers for individual layers or papers from other producers were used, which could have resulted in slight differences in the grammage of the corrugated board. Despite these minor differences, it was considered that the two sets of samples may be confronted, and a direct comparison of the test results of the samples will allow for the drawing of reliable conclusions.

**Figure 6:**
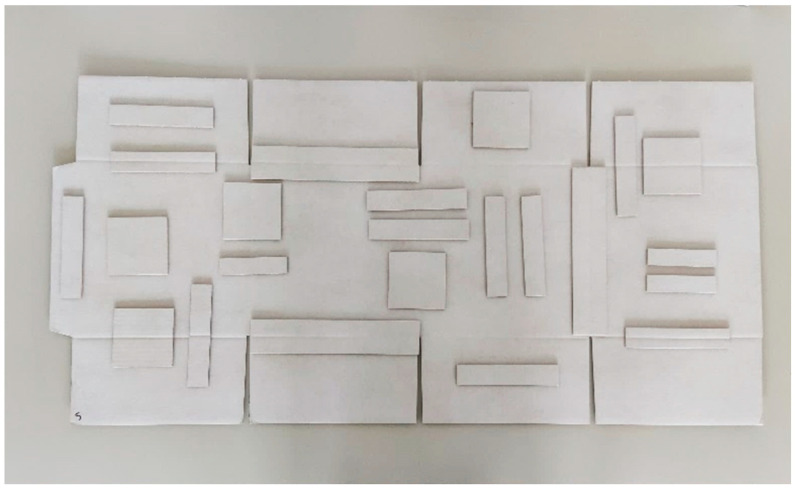
The box considered in this study before folding, with positions of carboard samples cut for tests.

The three boards were used to produce boxes, by both analog and digital technology, while cutting and making crease lines. The box design selected for this study was FEFCO code F201, with dimensions of 250 × 250 × 260 mm; see Figure 6. For each corrugated board, two samples of the materials were obtained. From six types of material boards (three corrugated boards from two production technologies), the samples for the ECT, SST, TST, and BNT were cut; see Figure 6. Moreover, to verify the influence of analog and digital crease lines on the mechanical parameters of corrugated board, the samples were cut from three regions: (i) crease-free region, (ii) crease lines, and (iii) perpendicular to crease lines. Some boards were also left to fold boxes for the BCT; see Figure 5.

For each of three regions, six samples were cut for a particular type of box sample. Therefore, a set of 108 (3 × 2 × 3 × 6) samples was prepared for the BSE System [49], which includes the ECT, SST, TST, and BNT. One set of samples for the BSE System requires seven specimens, one for the ECT, one for the SST, two for the TST (in the MD and CD directions) and two for the BNT (MD and CD directions).

In box compression tests, six samples were used for a particular type of box. Therefore, 36 (2 × 3 × 6) box samples were tested. In order to obtain a statistical representation of the corrugated board, we utilized six samples while testing the cardboards and six samples while testing the boxes.

### 2.5. Numerical Model of a Box

**Figure 7:**
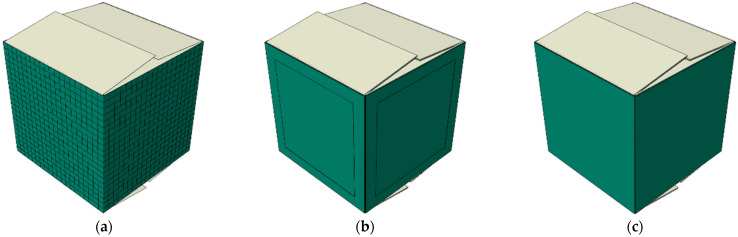
Numerical modelling of a box: (**a**) FE mesh, (**b**) model with two different material regions, and (**c**) model with single material region.

The numerical model was prepared using the commercial Abaqus Unified FEA software (Dassault Systemes SIMULIA Corp., Johnston, RI, USA) [62]. The model uses shell finite elements, S4, four-node, bilinear elements with four Gauss points. The mesh on the side walls is shown in Figure 7a. The flaps have no mesh and are not included in the calculations; they serve as a so-called display body. The orthotropic linear elastic model and the inelastic Hill material model embedded in Abaqus were used to simulate the corrugated board. Although the Hill model is not dedicated to material such as corrugated board, in which there is no symmetry in the compression and tensile behaviors, in the case of the dominance of compressive stresses that occurs in the BCT, it is sufficiently adequate. In order to obtain the correct material parameters from the laboratory test, the methodology based on numerical homogenization described in [27,28] was used.

Each vertical wall of the box model was divided into two partitions: (a) the edge area where the material models account for the crushing of the corrugated board and (b) the remaining areas where the material was not crumpled (see Figure 7b). In order to compare the results obtained with the model, which takes into account the crushing of the material in the vicinity of the vertical creasing lines, a packaging model was also built in which these effects were not taken into account (see Figure 7c).

In order to correctly model the progressive plasticization of the cardboard packaging, a nonlinear iterative analysis based on the Newton–Raphson procedure was used. In order to induce buckling from the plane of each vertical wall of the box, small imperfections were introduced into the model, which were determined in the linear perturbation buckling analysis.

## 3. Results

### 3.1. Corrugated Board Testing

**Figure 8:**
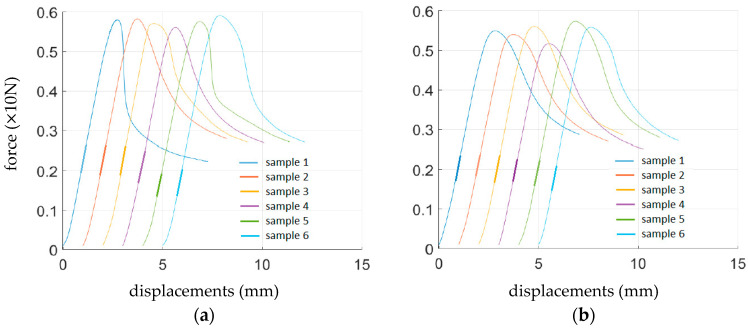
Bending stiffness test results from six samples of B370 corrugated board for each production technology: (**a**) analog and (**b**) digital crease lines.

The results from cardboard tests (see Section 2.1) for each sample (see Section 2.4) were averaged and are presented in Table 2. ECT is the edge crush test index (N/m), SST is the shear stiffness of corrugated board (Nm), TST_i_ is the torsion stiffness (Nm) in two directions (MD and CD, 1 and 2, respectively), and BNT_i_ is the bending stiffness (Nm) in two directions (MD and CD, 1 and 2, respectively). The results are presented for selected samples and testing protocols, namely, cardboard B370 and the bending stiffness test; see Figure 8 (the plots are horizontally shifted for clarity). The results summarized in Table 2 represent three types of corrugated boards, namely B360, B370, and B380, as well as different production technologies for crease lines, namely analog and digital. Moreover, the different samples were acquired from the produced box grids, that is, (i) samples from crease-free regions, (ii) samples with crease line along the sample, and (iii) samples with crease line perpendicular to the sample; see Figure 6. For each type of corrugated board, the ECT, SST, TST_1_, TST_2_, BNT_1_, and BNT_2_ parameters obtained from the BSE System are presented. For samples perpendicular to the crease, only the SST, TST_1_, and TST_2_ were undertaken; for the ECT, BNT_1_, and BNT_2_, the samples perpendicular to the crease would not produce beneficial results.

**Figure 9:**
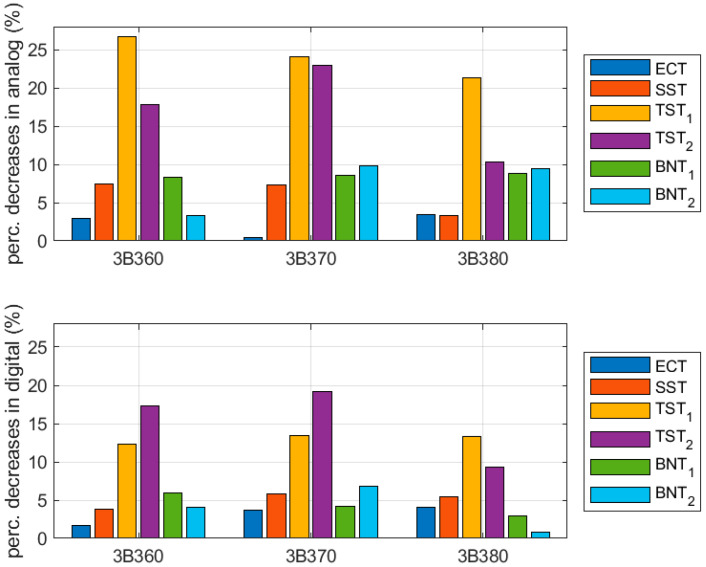
Bar plots of percentage differences between results for crease-free region samples and samples with a crease along the sample length.

The results for crease-free region samples and samples with the crease along the sample length were compared to verify the influence of the crease lines, in particular production technologies. The data are presented in Table 3 and Figure 9. The values shown are computed according to the following expression:

(1) $$P_{board1}=\left|100\%\;\cdot \left(1-\frac{S_{along\;crease}}{S_{no\;crease}}\right)\right|$$

where $S_{i}$ are the mean mechanical parameters presented in Table 2, with i=along crease for samples with the crease along the length and i=no crease  for samples with no crease.

**Figure 10:**
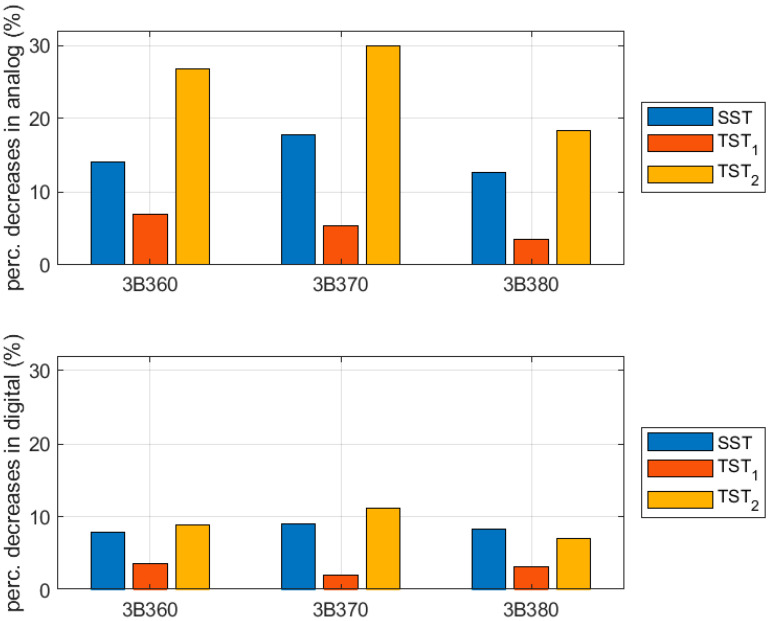
Bar plots of percentage differences between results for crease-free region samples and samples perpendicular to the crease.

The results for crease-free region samples and samples perpendicular to the crease were compared to verify the influence of the crease lines, in particular production technologies. The data are presented in Table 4 and Figure 10. The values shown are computed according to the following expression:

(2) $$P_{board2}=\left|100\%\;\cdot \left(1-\frac{S_{perp.\;\;to\;crease}}{S_{no\;crease}}\right)\right|$$

where $S_{i}$ are the mean stiffnesses presented in Table 2, with i=perp. to crease for samples perpendicular to the crease and i=no crease  for samples with no crease.

### 3.2. Compression Test of Corrugated Board Packaging

**Figure 11:**
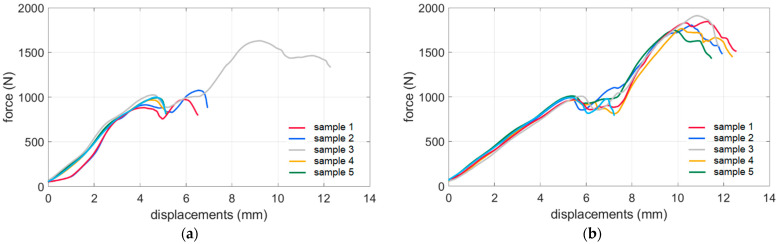
Box compression test results from six samples of B380 corrugated board for each production technology: (**a**) analog and (**b**) digital crease lines.

The results from the BCTs (see Section 2.2) for each sample (see Section 2.4) were averaged and are presented in Table 5. The results represent three types of corrugated boards, namely B360, B370, and B380, as well as different production technologies for the crease lines, namely analog and digital. Moreover, test curves for boxes made of B380 corrugated board are shown in Figure 11.

The results for boxes with analog crease lines and digital crease lines were compared to verify the influence of the crease lines on the compressive strength of the boxes due to the particular production technology. The data are presented in Table 6. The values shown are computed according to the following expression:

(3) $$P_{BCT}=\left|100\%\;\cdot \left(1-\frac{BCT_{digital}}{BCT_{analog}}\right)\right|$$

where $BCT_{i}$ is the mean compressive strength presented in Table 2, i=digital for samples with digital crease lines, and i=analog  for samples with analog crease lines.

The box tested had a so-called offset between the horizontal crease lines. This causes two peaks on the compression plots in typical force vs. displacement graphs; see Figure 11. The first is related to losing wall stability with greater height (its top contacts with the press first). In Table 5 and Table 6, the value of the second peak is shown in brackets.

### 3.3. Numerical Validation

Based on all mechanical measurements of corrugated board carried out in the BSE System, four material models were created: (i) corrugated board from analog-cut packaging, (ii) cardboard crushed by an analog cut and creasing process, (iii) corrugated board from digitally cut packaging, and (iv) cardboard crushed by a digital (hybrid) creasing process. All parameters (elastic and plastic) are summarized in Table 7.

**Figure 12:**
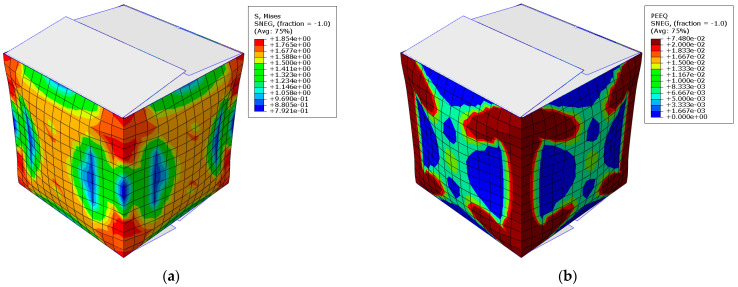
FE results in terms of: (**a**) Mises stresses and (**b**) equivalent plastic strains.

Numerical validation consisted of checking whether the A model, which takes into account the effect of crushing the corrugated board within 25 mm of all (vertical and horizontal) creasing lines, appears to be more precise than the traditional B model, in which these effects are ignored. Figure 12 shows an example map with Mises stresses (left) and plastic equivalent strains (right), which demonstrates the amount and location of plastic weakening. It is clearly visible that the progressive damage to the box (plasticization) starts and is concentrated in the corners of the packaging and propagates diagonally toward the center of the vertical walls.

The confrontation of the numerical results with the experimental results (see Table 8) provides justification that the introduction of modified material parameters in areas next to the creasing lines allow for a more precise estimation of the load capacity of the package in the static compression test.

In the numerical model, all four vertical walls were directly loaded (the displacement control approach was used); thus, the offset effect [5] was not captured. This simplification was used due to the small value of the shift, and therefore, only the second pick from the experimental curves was compared with the numerical predictions. Despite this simplification, the results obtained with the use of numerical model A do not differ from the experimental results by more than 4.19% (see Table 9).

## 4. Discussion

In Table 3 and Figure 9, the results for crease-free region samples and samples with a crease along the sample length were compared. It was shown that for all tests in the analog cases, i.e., the ECT, SST, TST_1_, TST_2_, BNT_1_, and BNT_2_, the samples lost from a few percentage points to up to 35% of their values. For the counterpart digital cases, the losses were usually meaningfully smaller, from a few percentage points to 31%.

Comparing the analog and digital crease line results in detail, the values for the ECT are similar while the values for the SST are a few percentage points lower (~2%) for digital crease lines. Further, the values for TST_1_ are much lower (9–13.5%) while the values for TST_2_ are a few percentage points lower (1–3%) for digital crease lines. Moreover, the values for BNT_1_ are also a few percentage points lower (3–5%), and the values for BNT_2_ are a few percentage points lower (2–4%) for digital crease lines. To sum up, in most cases, the samples with digital crease lines have lower decreases in the values of the mechanical parameters, if one compares the mechanical properties of no-crease samples and samples with creases along the sample length.

In Table 4 and Figure 10, the results for crease-free region samples and samples with creases perpendicular to the sample length were compared. It was shown that for the tests considered, i.e., the SST, TST_1_, and TST_2_ in the analog cases, the samples lost from a few percentage points up to 30% of their values. For the counterpart digital cases, the losses were usually meaningfully smaller, from a few percentage points to about 11%.

Comparing the analog and digital crease line results in detail, the values for the SST are 3–9% lower for digital crease lines. The values for TST_1_ are 1.5–3.3% lower while the values for TST_2_ are 11–18.8% lower for digital crease lines. To sum up, in all considered cases, the samples with digital crease lines have lower decreases of mechanical parameter values, if one compares the mechanical properties of no-crease samples and samples with creases perpendicular to the sample length.

While the samples made using the digital method presented lower decreases in their mechanical properties than those made by the analog method, it may be thought that the values are still small (1–5%). However, the values have an important impact on box compression strength. The main cost in producing packaging is the corrugated board itself. Therefore, employing the digital method can provide benefits in using lower quality and less expensive material, while still giving the box the strength required; it is less susceptible to crushing than more expensive and better quality materials. The cost of making the cardboard via the digital method is probably higher but remains more competitive due to the cheaper materials used in the final packaging.

In Table 5, the results of compressive strength tests for different crease lines and cardboard materials are presented. It may be observed that for the analog crease lines, the compressive strengths are between 0.96 and 1.21 kN, while for digital crease lines, the counterpart values are between 0.99 and 1.35 kN. The percentage differences are presented in Table 6. For B360 boxes, the difference was 18%; in other words, on average, the compressive strength of the box with digital crease lines was 18% higher than for boxes with analog crease lines. For B370 and B380 corrugated boards, values of 8.4% and 2.8% were obtained, respectively. This also proved that digital crease lines reduce the compression strength of the box to a lesser extent than analog crease lines.

For B380, the analog cardboard had a grammage of about 30 g more in comparison with the digital cardboard, but the box made of material obtained from digital technology still had higher strength (2.8%). It was also observed that the drop of the strength curve after the first peak in digital technology products is much smaller than in analog technology. The percentage difference between the analog and digital first peak is 2.8%, increasing to about 10% for the second peak (see Table 6).

To the authors’ knowledge, similar studies analyzing the effects of analog and digital creasing lines on the performance of corrugated board and boxes have not been published in the scientific literature. Therefore, the results presented here are unique and cannot be compared with the previous research. The results evidently demonstrate that digital technology is not only more repeatable, but also causes far less damage to the structure of corrugated board than analog technology.

Similarly, the authors have not been able to find adequate research in the literature in which numerical models take into account the effect of the crushing of the corrugated board with respect to analog and/or digital creasing. From the research and calculations performed here, an interesting conclusion can be drawn. In the case of model B, which does not take into account the crushing effect for box samples with digital creasing lines, the difference between prediction and the experiment was only 1–2%. However, when the crushing effects were not taken into account in the boxes with analog creases, the difference reached 13.7%. This observation makes it reasonable to take into account regions where the material is evidently different (crushed) in order to correctly model the behavior of the packaging. As creased material measurements are not subject to a common standard, it remains to take into account the drop in load capacity with a correction factor. An alternative is to produce the packaging while using digital tools to gain 10–13% of the load capacity of each package, at the expense of implementing a new technology in the production plant.

## 5. Conclusions

This study verified the influence of two types of creasing line technologies, analog and hybrid digital, while applying digitally driven mechanical crease rules on the mechanical parameters of corrugated board and packaging. The research was carried out on corrugated board samples with edge crush resistance, shear stiffness, torsional stiffness, and bending stiffness measured for three types of three-layer board with different grammages. The same materials were used to test the boxes to determine and compare their compressive strength. All the measured mechanical properties of the corrugated board were used to build the correct constitutive models for both the undamaged and the crushed material for numerical analysis, which was performed to verify the packaging behavior during physical load-bearing tests.

First, the laboratory data were analyzed to understand the difference in the deterioration of the mechanical properties of corrugated board and box when analog or digital creasing lines are used. It turned out that the use of digital crease lines can be beneficial, as they have a reduced impact on the shear and torsional stiffness of the cardboard. Although the decrease in bending stiffness is less, it is still noticeable. The drops in the edge crush tests are negligible. Likewise, boxes with digital crease lines are stronger compared to analog crease lines if box compression is analyzed. Producing crease lines by digital technology does not crush the corrugated board, due to which its material properties are higher and also give higher load-bearing capacity to boxes with such crease lines.

Numerical simulations were then carried out to understand the impact of crushing in the vicinity of the creased edges of the packages on its load-bearing capacity. The most important conclusion that emerged after the validation while using two numerical models is the fact that only boxes made of corrugated board with hybrid creasing can be analyzed using a model that does not take into account the effects of cardboard crushing—in this case, the estimation error was within 2.02%. However, in order to correctly estimate packaging with analog creases, it is necessary to take into account the effects of the crushing of the corrugated board as a result of the analog finishing process and to model two material regions (intact and deteriorated due to creasing). In the case of boxes with analog creases, the numerical model, which did not take into account the effect of crushing, generated results as much as almost 12% higher than the model, which took these effects into account, which means that 12% of the load capacity is lost by clinging to traditional packaging finishing techniques. To sum up, modern methods of packaging finishing can not only help producers of corrugated boxes to digitalize their production, but can also reduce the weight of their products and thus save more trees that are for the common good.

## Figures and Tables

**Table 1:** Grammage and thickness of all cardboard samples.

Cardboard	Grammage Analog (gr/m^2^)	Thickness Analog (mm)	Grammage Digital (gr/m^2^)	Thickness Digital (mm)
B360	368.22	2.79	366.06	2.80
B370	393.50	2.83	393.28	2.78
B380	413.78	2.76	385.00	2.74

**Table 2:** Averaged test results for corrugated board with different types of cardboard, production technology, and sampling region.

Cardboard	Crease Lines	Sample	ECT (N/mm)	SST (Nm)	TST_1_ (Nm)	TST_2_ (Nm)	BNT_1_ (Nm)	BNT_2_ (Nm)
B360	analog	no crease	5.038	1.098	0.722	0.599	1.642	2.898
B370			4.828	1.438	0.893	0.688	2.268	4.363
B380			5.066	1.198	0.798	0.548	1.985	3.551
B360	digital	no crease	5.079	1.068	0.705	0.596	1.637	3.134
B370			4.826	1.428	0.891	0.686	2.254	4.255
B380			4.543	1.219	0.768	0.539	1.936	2.818
B360	analog	along crease	4.888	1.017	0.529	0.492	1.506	2.995
B370			4.808	1.333	0.678	0.530	2.073	3.935
B380			4.890	1.159	0.628	0.492	1.810	3.216
B360	digital	along crease	4.992	1.028	0.618	0.493	1.539	3.008
B370			4.648	1.345	0.772	0.555	2.160	3.967
B380			4.360	1.152	0.666	0.489	1.880	2.794
B360	analog	perp. to crease	-	0.944	0.672	0.438	-	-
B370			-	1.182	0.844	0.482	-	-
B380			-	1.047	0.770	0.447	-	-
B360	digital	perp. to crease	-	0.984	0.680	0.543	-	-
B370			-	1.299	0.873	0.610	-	-
B380			-	1.117	0.744	0.502	-	-

**Table 3:** Percentage differences between results for crease-free region samples and samples with a crease along the sample length.

Cardboard	Crease Lines	ECT (%)	SST (%)	TST_1_ (%)	TST_2_ (%)	BNT_1_ (%)	BNT_2_ (%)
B360	analog	2.99	7.43	26.7	17.7	8.26	3.34
B370		0.42	7.29	24.0	22.8	8.62	9.82
B380		3.48	3.29	21.3	10.2	8.81	9.42
B360	digital	1.71	3.76	12.3	17.2	5.99	4.04
B370		3.68	5.81	13.3	19.1	4.18	6.76
B380		4.03	5.47	13.3	9.28	2.90	0.85

**Table 4:** Percentage differences between results for crease-free region samples and samples perpendicular to the crease.

Cardboard	Crease Lines	SST (%)	TST_1_ (%)	TST_2_ (%)
B360	analog	14.1	6.93	26.8
B370		17.8	5.41	29.8
B380		12.6	3.51	18.3
B360	digital	7.93	3.65	8.89
B370		9.01	2.05	11.2
B380		8.33	3.21	7.02

**Table 5:** The averaged results from box compression testing for different cardboards and production technologies.

Cardboard	Crease Lines	Compressive Strength (kN)
B360	analog	1.145
B370		1.208
B380		0.963 (1.631)
B360	digital	1.350
B370		1.310
B380		0.990 (1.814)

**Table 6:** Percentage differences between compressive strength of boxes with analog and digital crease lines.

Cardboard	Compressive Strength (%)
B360	18.0
B370	8.43
B380	2.77 (10.01)

**Table 7:** Material parameters in four different constitutive models. The subscript “d” refers to a digital finishing process while “a” refers to an analog finishing process.

Parameter	$\mathrm{mat}\;A_{d}\;$	$\mathrm{mat}\;B_{d}$	$\mathrm{mat}\;A_{a}$	$\mathrm{mat}\;B_{a}$
$E_{11}$ (MPa)	1831	1803	1811	1617
$E_{22}$ (MPa)	850	916	868	928
$\nu_{12}$ (-)	0.42	0.41	0.42	0.38
$G_{12}$ (MPa)	3362	2685	3566	2913
$G_{23}$ (MPa)	2.34	3.27	2.35	3.05
$G_{13}$ (MPa)	3.05	3.68	2.64	3.73
$\sigma_{0}$ (MPa)	1.74	1.78	1.71	1.76
$R_{11},\;R_{22},\;R_{33}$	1, 0.73, 0.73	1, 0.75, 0.75	1, 0.52, 0.52	1, 0.62, 0.62
$R_{12},\;R_{23},\;R_{13}$	0.46, 0.46, 0.46	0.5, 0.5, 0.5	0.36, 0.36, 0.36	0.41, 0.41, 0.41

**Table 8:** Experimental results versus estimation using two different numerical models. Model A with the effect of crushing next to the creasing line and Model B without this effect.

Cardboard	Crease Line	Compressive Strength (kN)		
		Experiment		
B360	analog	1.145	B360	analog
B370		1.208	B370	
B380		1.631	B380	
B360		1.350	B360	
B370	digital	1.310	B370	digital
B380		1.814	B380	

**Table 9:** Percentage difference between experimental results and estimations using two different numerical models as well as a difference between the BCT computed using Model A and Model B.

Cardboard	Crease Line	BCT Estimation Difference (%)		
		Experiment vs. Model A		
B360	analog	2.80	B360	analog
B370		−3.78	B370	
B380		−2.32	B380	
B360		−2.97	B360	
B370	digital	−1.71	B370	digital
B380		−4.19	B380	

## Data Availability

The data presented in this study are available on request from the corresponding author.
